# Extracts from the leaves of knotweeds (*Reynoutria* spp.) have a stimulating effect on the germination and initial growth of wheat grains

**DOI:** 10.1007/s00114-024-01946-0

**Published:** 2024-11-05

**Authors:** Božena Šerá, Pratik Doshi, Lubomír Věchet

**Affiliations:** 1 Department of Environmental Ecology and Landscape Management, Faculty of Natural Sciences, Comenius University Bratislava, Ilkovičova 6, 84215 Bratislava, Slovakia; 2 Crop Research Institute, Drnovská 507/73, 161 06 Praha 6 - Ruzyně, Czech Republic

**Keywords:** Alcoholic extract, Plant growth, *Polygonum*, Seed priming, Seedling vigor index

## Abstract

Knotweed (*Reynoutria* sp.) plants are known in the world mainly as invasive plants. However, it is known that their rhizomes or leaves contain secondary metabolites with biological activity. Our goal was to determine which of the three knotweed plants (*Reynoutria japonica*, *Reynoutria × bohemica*, and *Reynoutria sachalinensis*) is most suitable for seed growth stimulation. We tested alcoholic extracts of all three knotweed species by seed priming method on wheat germination and seedling characteristics, when 12 measured characteristics were monitored. Extracts from all three species of *Reynoutria* sp. generally showed an improvement in wheat germination and growth compared to the control. *R.* × *bohemica* appears to be the best source for stimulating wheat growth, as seedling vigor indexes I and II, R/S dry weight, shoot and seedling lengths, root, shoot, and seedling dry weights were significantly different (ANOVA, Duncan’s test, *α* < 0.05). The plants *Reynoutria* spp. seem to be possible sources for the protection and stimulation of agriculture crops.

## Introduction

Invasive species are non-native species that are generally introduced due to human-caused global environmental changes, resulting in shifts in native ecosystems (Pejchar et al. 2009). Furthermore, invasive species are responsible for tremendous costs to the global economy (Cook et al. 2007). Over the past few decades, invasive plant species have posed several threats to native biodiversity, ecosystem services, environmental quality, and human health (Rai and Singh 2020). One such invasive plant species that is common and has dramatically changed landscapes in the past and continues to do so in the present is knotweed.

Knotweeds are perennial herbs that create striking shrub-like growths. In Central Europe, three main knotweed species are commonly found: *Reynoutria japonica* Houttuyn (syn. *Fallopia japonica* (Houttuyn) Ronse-Decraene), commonly known as Japanese knotweed; *Reynoutria sachalinensis* (F. Schmidt) Nakai (syn. *Fallopia sachalinensis* (F. Schmidt) Ronse-Decraene), commonly known as Giant knotweed; and *Reynoutria* × *bohemica* Chrtek and Chrtková (syn. *F.* × *bohemica* (Chrtek and Chrtková) J.P. Bailey), commonly known as Bohemian knotweed.

*R. japonica* ranks as “100 of the world’s worst invasive alien species in the world” (Lowe et al. 2000). After its introduction, *R. japonica* was found to alter the diversity of plant and invertebrate species in natural riparian habitats (Gerber et al. 2008). For example, under controlled conditions, Abgrall et al. (2018) found that knotweed rhizome extracts showed effects on soil fauna, namely, the nematodes and Collembola, at different trophic levels. Renčo et al. (2021) also found similar results in the ruderal habitats of the Tatra national park in Slovakia. A detailed effect of *Reynoutria* species on the different plant, insects, and animal species can be found in the review of Lavoie (2017).

*R. sachalinensis* is a traditional medicinal plant mainly distributed in China, Korea, and Japan (Zhang et al. 2005). It was first introduced to Europe in the nineteenth century and, since then, has rapidly established itself as an invasive species. Despite its traditional use in Asia, *R. sachalinensis* has mild negative effects on the species richness of various plant species (Tokarska-Guzik et al. 2006), such as lower species richness among different riparian species (Urgenson et al. 2014) and a reduced germination rate in *Calamagrostis epigeios*, *Lepidium sativum*, and *Urtica dioica* (Moravcová et al. 2011). However, according to a report by the European Food Safety Authority (EFSA 2015), there is a knowledge gap on the long-term negative effects of *R. sachalinensis* on the environment. *R.* × *bohemica*, a hybrid found among *Reynoutria* spp., spreads faster than its parent species, resulting in dense and tall stands. Consequently, the richness of plant species is severely reduced in its vicinity, modifying the structure of the plant community (Levačić et al. 2023). The allelopathic effects of *R.* × *bohemica* impact the seedling germination of other plant species, along with the absence of shade-tolerant species (Moravcová et al. 2011).

Management of knotweeds has always been a priority for land managers due to their rapid spread, which threatens biodiversity (Cottet et al. 2020). Previously, land managers relied on their own experience and advice from fellow managers without scientific knowledge or understanding to control knotweeds (Cottet et al. 2020). For example, in France, managers have termed knotweeds “unmanageable” and “foreign” in less invaded areas (Rouifed et al. 2018). Various management strategies have been proposed to control knotweeds, including cutting, mowing, pulling, digging, tarping, burning, grazing, planting, salting, and using herbicides or biological control (Dusz et al. 2021).

Ecosystem services are defined by the Millennium Ecosystem Assessment (2005) as “the benefits people obtain from ecosystems.” These benefits can be provisioning or tangible, such as wood, fiber, food, and timber, or intangible, as defined under cultural ecosystem services, which include spiritual and religious benefits. Ecosystem services are receiving increasing attention as the human population grows and becomes more dependent on nature for its needs (Costanza et al. 1997; Millennium Ecosystem Assessment 2005). This is why ecosystems are managed by humans according to their needs. Knotweeds have been providing various ecosystem service provisioning services. For example, resveratrol, a polyphenolic nutraceutical found in knotweed species, has been shown to be beneficial in the field of medicine against diabetes mellitus, obesity, metabolic syndrome, hypertension, Alzheimer’s disease, stroke, cardiovascular diseases, kidney diseases, inflammatory diseases, rhinopharyngitis, and cancer (Singh et al. 2019; Rauf et al. 2017).

In addition to medicine, knotweeds have also been researched for plant protection and growth. For instance, *R. sachalinensis* has been investigated for its effects against powdery mildew (Daayf et al. 1995, 1997; Konstantinidou-Doltsinis et al. 2006). *Reynoutria* spp. are not limited to the control of powdery mildew; Nawrot-Hadzik et al. (2019) also tested the antibacterial activity of Reynoutria sp. acetone extracts against dominant caries pathogens such as *Streptococcus mutans* and other alternative pathogens. Resveratrol has been found to protect grapevines against *Botrytis cinerea* (Adrian and Jeandet 2012) and tobacco against *Ralstonia solanacearum *in vitro and in vivo (Chen et al. 2016), while its external application has been shown to improve postharvest disease resistance in fruits (Ureña et al. 2002).

Knotweeds are also found to have a positive stimulating effect, especially on the growth of economically important crop plants. For example, Karavaev et al. (2007) found that spraying barley plants with extracts of *R. sachalinensis* increased the photosynthetic activity, number of stems, and yield. Several other studies have tested the effects of knotweeds on seed germination and plant growth in wheat (Anžlovar et al. 2020; Šerá et al. 2015) and Tartary buckwheat (*Fagopyrum tataricum*) (Anžlovar and Anžlovar 2024).

Wheat is the second-most cultivated and widely consumed crop globally (Igrejas and Branlard 2020). It is cultivated on 220 million hectares worldwide and is known to provide energy and essential nutrients to humans (Nyaupane et al. 2024). Total annual average wheat production has increased by 2% to accommodate the future needs of the entire population (Šafář et al. 2010). However, due to abiotic stress, global yields are declining, and global warming is facilitating the rise of various pests and pathogens that hinder wheat yield by inducing resistance to different chemical pesticides that are harmful to the environment.

In this study, we present a potential new outlook on how invasive knotweeds can be exploited under provisional ecosystem services and their use in agroecological services, specifically as plant stimulants for seed germination. We tested the alcoholic extracts of three knotweed species as plant stimulants on wheat grain germination and seedling growth.

## Material and methods

### Extract preparation

Leaves from each individual species (*R. japonica*, *R. sachalinensis*, and *R.* × *bohemica*) were collected from three different populations in the South Bohemia region of the Czech Republic, characterized by good vitality; the leaves were not damaged by insects. The leaves were immediately dried for 62 h at 50 °C, after which the three samples were mixed. The alcoholic extract was prepared from 100 g of crushed dry matter and 850 mL of 20% ethanol with intensive shaking for 5 h, followed by filtering of the sediment. The choice of concentration is based on data from previous experiments (Vrchotová et al. 2010; Vrchotová and Šerá 2008). In this way, three extracts were prepared, each corresponding to a different type of knotweed (*R. japonica*, *R.* × *bohemica*, *R. sachalinensis*). The extracts were stored in the dark at 6 °C.

### Grain priming

Common wheat grains (*Triticum aestivum* L. cv. Kanzler) were obtained from the collections of the Crop Research Institute in Prague. Only healthy and undamaged grains were used visually. The grains were soaked in 500 mL of plant extracts and gently shaken with a rotary shaker at room temperature (24 h, 20 °C). The liquid was then filtered and the grains were dried and stored in the dark at room temperature for 2 weeks. A total of 150 wheat grains were used for each type of pretreatment. The control sample (150 grains) was pretreated with distilled water.

### Germination test

Five plastic Petri dishes (diameter 9 cm, each containing 3 sheets of KA quality filter paper, 6 mL of distilled water, and 30 grains per dish) were used for one treatment (150 grains per treatment). The dishes were stored in an incubator (in darkness, at a temperature of 20 °C) for 5 days. A grain was considered germinated if the sprout was at least 1 mm long. The number of germinating grains, the length of the seedling shoot (in mm), and the longest length of the seedling roots (in mm) were monitored on day 5. The seedlings of each Petri dish (*n* = 30) were then harvested, dried, and their weight per dish (in grams) was determined using a laboratory scale (balance sensitivity 0.001 g) after drying.

The length of the root (in mm) was calculated as the average of one seedling from five dishes for one treatment. The lengths of the shoot (in mm) and seedling (in mm) were calculated similarly. The weight of the dried root (in mg) was calculated as the average weight per dish from one treatment. The weights of the dried shoot (in mg) and dried seedling (in mg) were calculated in the same manner. The following characteristics of seed germination and early seedling growth were calculated according to the methodology outlined by Šerá (2023): seed germination (%), germination index, root length (mm), shoot length (mm), seedling length (mm), seedling vigor index I (mm), seedling vigor index III (mg), root-to-shoot length ratio (R/S_length), and root-to-shoot dry weight ratio (R/S_dry_weight).

### Statistical analyses

The data obtained were analyzed using the statistical package (StatSoft, Inc 2013) at a significance level of 0.05. One-way analysis of variance (ANOVA) and post hoc comparison (PHC) were performed. For PHC, Duncan’s test was used to evaluate the differences among the plant extracts in relation to wheat germination and early growth. The dependent variables were wheat characteristics, while the independent categorical variable was knotweed extract (*R. japonica*, *R.* × *bohemica*, *R. sachalinensis*).

## Results

ANOVA confirmed a significant difference among the extracts used (*p* < 0.00025). Differences among the tested plant extracts in relation to wheat germination and early growth (PHC test) were observed in almost all monitored characteristics (Table 1). Only for seed germination and the germination index were no significant differences found. The highest values of these characteristics were measured in the control samples (Table 1). In 8 of 12 characteristics, the highest values were found after treatment with an extract of *R.* × *bohemica*, while in two characteristics, the highest values were observed after using *R. japonica* (Table 1).

**Table 1:**
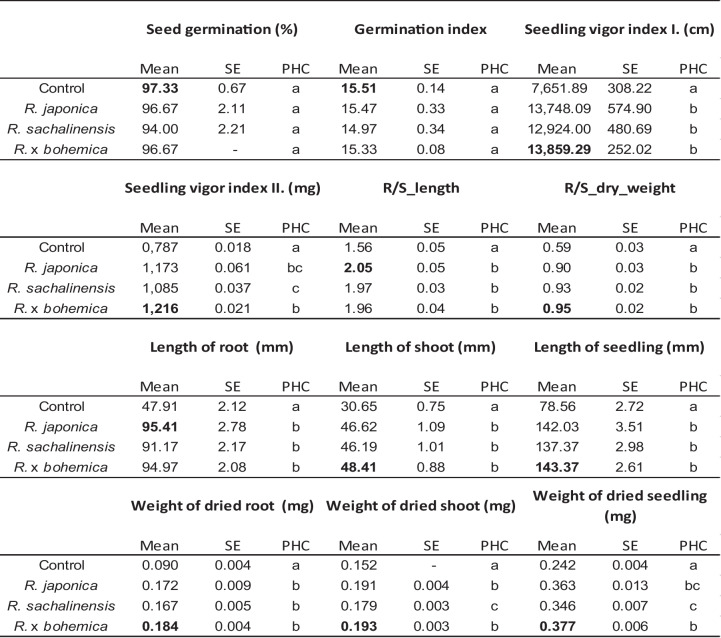
Overview of the measured values of the detected characteristics in three knotweed (*Reynoutria* spp.) extracts. The highest measured values are marked in bold; *SE* standard error, *PHS* result of Duncan’s test, where different letters indicate significant differences

## Discussion

As studies have shown (Table 1), except for seed germination and germination index, all other seedling characteristics were significantly higher compared to the control. Among the three knotweeds, *R.* × *bohemica* exhibited the highest increase in seedling characteristics. Previous studies have tested the allelopathic effects of *Reynoutria* sp. extracts on germination and seedling development. For example, Borovaya et al. (2020) found that *R. japonica* extract not only was an effective antifungal agent against *Septoria glycines* but also promoted the growth of three crop species. *Triticum aestivum*, *Hordeum vulgare*, and *Glycine max*. This could be attributed to the different allelochemicals found in *R. japonica* and their effects on various plant species. Similarly, Zorikova (2011) found that the water extract of *R. japonica* had a stimulating effect on seed germination and seedling characteristics of *Tagetes erecta*. Our results contradict those of Novak et al. (2018), who found a strong negative allelopathic effect in the water extract of the entire *R. japonica* plant material in oats (*Avena sativa*), oilseed rape (*Brassica napus* subsp. *oleifera*), and sunflower (*Helianthus annuus*). Such negative effects of the rhizome extracts of all *Reynoutria* spp. were also observed by Vrchotová and Šerá (2008) in the seeds of white mustard (*Leucosinapis alba*). This could be attributed to the chemical composition in the above-ground and below-ground parts of *Reynoutria* spp.

In our study, we also found that *R. sachalinensis* showed significantly better wheat growth characteristics compared to the control. This could be attributed to three flavonoids: quercetin-3-O-α-L-arabinofuranoside, quercetin-3-O-α-D-galactopyranoside, and quercetin-3-O-β-D-glucuronopyranoside (Zhang et al. 2005), which have antioxidant activity. On the contrary, Koce and Šoln (2017) reported phytotoxicity in radish (*Raphanus sativus*) and maize (*Zea mays*) when treated with aqueous extracts (0.5% and 5% (w/v)) of *R. japonica* and *R.* × *bohemica* knotweeds. They found that the extracts did not interfere with germination, but inhibited root growth at later stages of early growth. In addition, they observed decreased catalase activity, increased lipid peroxidation, total antioxidative capacity, and no specific effect on guaiacol peroxidase. Mikulic-Petkovsek et al. (2022) also found similar results in perennial ryegrass, stating that this phytotoxic effect of *R. japonica* could be due to phenolic groups such as hydroxycinnamic acids and flavanols.

Similar conclusions were drawn by Karavayev et al. (2010) on bean seed germination. Šerá (2012) tested soil substrates contaminated with the three species of *Reynoutria* in our study and evaluated their germination properties in two crop plants (*Leucosinapis alba*, *Brassica napus*) and two weed species (*Chenopodium album* agg., *Echinochloa crus-galli*). She found contrary results compared to our study; all three knotweed species significantly reduced germination in *L. alba*. Meanwhile, in *B. napus*, only *R.* × *bohemica* did not have a significant effect on seed germination, while it yielded the best results on seed growth in our study (see Table 1). She also found that none of the *Reynoutria* species exerted a significant negative effect on *C. album* agg. or *E. crus-galli*, which is consistent with our results for wheat (see Table 1).

We found similar results to those reported by Parepa et al. (2012) with respect to *R.* × *bohemica*, as they also did not find any negative effects of *R.* × *bohemica* leachate on seed germination and seedling characteristics of nine different plants, namely, two grasses (*Lolium perenne*, *Poa trivialis*) and seven forbs (*Filipendula ulmaria*, *Geranium robertianum*, *Geum urbanum*, *Glechoma hederacea*, *Silene dioica*, *Symphytum officinale*, and *Urtica dioica*). Based on their results, Parepa et al. (2012) developed the hypothesis that the extract/leachate of *Reynoutria* spp. alters the soil microbial community, facilitating better seed germination conditions. However, in our research, we did not perform tests to check for changes in the soil microbial community.

Although Vrchotová and Šerá (2008) assumed that the content of catechins and stilbenes differed between the three species of knotweed and that this could influence their allelopathic effects, it was Inoue et al. (1992) who tested the acetone extracts of the rhizomes of *R. sachalinensis* on the seeds of *Lactuca* spp., *Amaranthus viridis*, and *Phleum pratense*, finding that emodin and physcion, compounds of anthraquinone, were responsible for inhibiting the growth of radicle and hypocotyl in the treated seeds. However, two anthraquinone glucosides, namely, emodin-1-O-β-D-glucoside and physcion-1-O-β-D-glucoside, even at a concentration of 200 ppm, did not show phytotoxic effects on lettuce seeds.

An interesting case was presented by Šoln et al. (2021), who tested the inhibitory effects of methanol extracts from the rhizomes of *R. japonica* and *R.* × *bohemica* against radish germination and root growth. They found that a mixture of phenolic compounds from the rhizomes of both weed species was more damaging than individual phenolic compounds such as resveratrol, epicatechin, and emodin; this was also confirmed by Serniak (2016). Furthermore, the antioxidative capacity increased in seedlings exposed to 0.6 mg/mL of resveratrol and emodin (Šoln et al. 2021, 2022).

Regarding the different allelopathic effects of *Reynoutria* spp., we hypothesize that there could be single compound or combinations of different compounds (e.g., resveratrol, emodin-1-O-β-D-glucoside, physcion-1-O-β-D-glucoside, emodin, physcion, quercetin-3-O-α-L-arabinofuranoside, quercetin-3-O-α-D-galactopyranoside, quercetin-3-O-β-D-glucuronopyranoside) that could potentially trigger or suppress the germination and growth of different plants. Concentrations of these compounds may play a vital role in regulating plant germination and growth characteristics.

Moreover, it is essential to understand the genetic diversity among different plants as they can react to these compounds/metabolites differently. Although our research did not test the presence or concentrations of these compounds, it could open new avenues of research by narrowing down chemical compounds that can be used as “plant stimulants” of botanical origin in agriculture. This approach will help reduce the reliance on harmful chemicals and promote sustainable agricultural practices. Furthermore, it will encourage land managers to view *Reynoutria* spp. as an “opportunity under ecosystem services” rather than merely an invasive plant. Another promising area for future research is to identify how the soil microbial community is altered by compounds present in *Reynoutria* spp., which may better facilitate seed germination and plant growth. We recommend further testing of these botanically derived compounds for seed germination, seedling growth, and/or plant protection purposes.

## Conclusion

The extract obtained from the three *Reynoutria* spp. (*R. japonica*, *R.* × *bohemica*, *R. sachalinensis*) produced a noticeable biostimulant effect on wheat grains. Individual water extracts of dried leaves of *Reynoutria* spp. showed no negative effects on grain germination and initial growth parameters in wheat during laboratory tests. In fact, all treatments improved seedling growth. *R.* × *bohemica* was found to be the most effective treatment, as significantly higher values were observed in the seedling vigor index I, the seedling vigor index II, the root-to-shoot dry weight ratio, the length of the shoot, the length of the seedling, the weight of the dried root, the weight of the dried shoot, and weight of dried seedling. Considering the distribution of these three *Reynoutria* spp. in Europe, they could serve as potential raw materials for botanically derived plant stimulants for sustainable agriculture.

## Data Availability

All data generated and analyzed during this study are available from the corresponding author upon reasonable request.
